# Frailty is an independent predictor of incident dementia: Evidence from the English Longitudinal Study of Ageing

**DOI:** 10.1038/s41598-017-16104-y

**Published:** 2017-11-16

**Authors:** Nina T. Rogers, Andrew Steptoe, Dorina Cadar

**Affiliations:** 1 Department of Epidemiology and Public Health, University College London, London, UK; 2 Department of Behavioural Science and Health, University College London, London, UK

## Abstract

The aim of this study was to determine whether frailty in older adults is associated with the risk of subsequent dementia. A total of 8,722 older adults from the English Longitudinal Study of Ageing were followed-up every two years until they reported a diagnosis of dementia, died, or were right censored. Frailty was defined using a frailty index comprised of 47 health deficits. To test if cognitive function influences the relationship between frailty and incident dementia, the analyses were repeated according to lower or upper three quartiles of baseline cognitive function. Competing risks regression and Cox proportional hazard models were used to evaluate whether the degree of baseline frailty was associated with incident dementia. Compared with non-frail participants, pre-frail (HR: 1.51 95%CI [1.12–2.02]) and frail participants (HR: 1.73 95%CI [1.22–2.43]) had a higher risk of developing dementia, after adjustment for covariates. The association between frailty and incident dementia was significant for adults in the upper three quartiles of global cognitive function (HR: 3.48 95%CI [1.98–6.12]), but not for adults who were in the lowest quartile of cognitive function (HR: 1.13 95%CI [0.74–1.71]). Frailty should be monitored alongside cognitive functioning when assessing risk factors for dementia in older adults.

## Introduction

Around 800,000 people were estimated to be living with dementia in the United Kingdom (UK) in 2016, costing the UK economy approximately £26 billion per year^1^. These numbers are projected to double by 2040, primarily as a result of population ageing^1^. No drug treatment has been proven to modify dementia, and there is an urgent need to develop cost-effective strategies that will reduce the risk and delay the onset of dementia. Advancing age is consistently reported as being a major risk factor for dementia^2–4^, however it is noteworthy that individual risk of developing dementia varies greatly, even among people of the same age. Recent evidence suggests that dementia might be best understood by considering the accumulation of different health problems in older age, often described as frailty^5^. Frailty is a typically progressive condition of older adults that is characterised by increased vulnerability to minor stressors and loss of physiological reserve^6^. Frailty is also associated with adverse health outcomes including disability, hospitalisation, falls and death^6–8^. It has been implicated as a potential risk factor for the development of dementia, but the findings of different studies have been inconsistent^5,9–13^. Frailty was found to independently predict overall dementia over a 3.5 year follow-up period in a study of older Italian men and women aged 65 to 84 years^14^. A separate study demonstrated that frail compared to healthy individuals were 8 times more likely to have dementia and almost eight times more likely to be cognitively impaired^13^. Other studies have concluded that frailty *per se* is not associated with dementia, but that associations between the two could be explained by the involvement of cognitive ability measures in both frailty and dementia^15,16^. A study by Gray and colleagues reported that frailty was only associated with all-cause dementia in those with higher cognitive functioning scores^15^, contrary to this an unrelated study only found an association between frailty and incident dementia in individuals who were cognitively impaired^16^. These inconsistencies may be due to differences in how frailty, cognitive function and dementia are measured across different studies, but it is also noteworthy that competing threats to survival have not been taken into account in these studies. Frailty is associated with reduced survival^17,18^ and many of the risk factors associated with dementia also predict an earlier death^19^. When studying the relationship between frailty and dementia, it is therefore important to consider the fact that the cumulative incidence of death differs significantly between the individuals exhibiting different degrees of frailty. To put this more explicitly, many participants who might have had the most severe risks of developing dementia are likely to have died before any dementia diagnosis. Studies examining dementia do not always explicitly account for the competing risk of death^20^ even though failure to do so has been shown to overestimate the level of risk associated with different health outcomes^21^. In this study, we investigate degrees of frailty severity and subsequent incidence of dementia over a 10-year period, accounting for death as a competing risk. In addition, we investigate whether baseline cognitive function might affect the relationship between frailty and development of dementia.

## Methods

### Study population

The English Longitudinal Study of Ageing (ELSA) is an extensive multidisciplinary panel study that began in 2002 (wave 1). It involves a representative sample of the English population comprised of individuals aged 50+. ELSA collects information on the health, financial circumstances, lifestyle, behaviour and well-being of older adults. Further details about ELSA are published elsewhere^22^. Participants are interviewed in their homes every two years by a trained interviewer and data is collected via a computer-assisted interview. The National Health Service Health Research Authority granted ethical approval for all seven waves of ELSA.

#### Exposure

Frailty was defined using a frailty index, a commonly used construct that has been validated in large epidemiological trials^6,23^. A Frailty Index (FI) was generated by using the criteria of Rockwood and colleagues^24,25^. The FI included 47 variables that encompassed a range of states, conditions and physiological systems which included mobility, disability (activities of daily living and instrumental activities of daily living), self-rated general health, eyesight, hearing and chronic diseases including cardiovascular diseases and depressive symptoms^25^ (Table S1). The criteria for deficit inclusion into the FI was as follows: had a prevalence of at least 1%, had less than 5% missing data, did not saturate too early (the prevalence of the deficit does not attain 100% before older age), and are associated with adverse health outcomes^24^. The FI becomes unstable if there are too few health deficits being considered, however the inclusion of at least 30 deficits has been shown to predict adverse health outcomes without significant loss of predictive validity^5^. Importantly, when deficits are selected at random from a larger sample of deficits to derive a FI, there is no significant effect on the predictive power of FI on death or development of dementia provided that the number of deficits being considered are sufficient (> = 30). This suggests that it is the number rather than the nature of the health deficits that enables the FI to predict adverse outcomes^26^. Participants were therefore included in the sample if they had complete data for at least 30/47 deficits^24^. Each variable provided an equal contribution to the frailty score and was assigned a score of 1 for each deficit that was present and a score of 0 for every deficit that was absent. A score between zero and one was given to deficits that could be present to some limited or partial extent. The FI was derived by calculating the mean number of health deficits present in participants that had valid measures on at least 30 health deficits. Hence, totalling the number of deficits and dividing them by the number of deficits assessed in that individual derived the frailty index. The FI was categorised into three groups, according to the degree of frailty, the parameters of which have been documented in other studies as non-frail: (FI ≤0.08); pre-frail (0.08 >FI ≤0.25) and frail (FI >0.25)^8^.

#### Missing data

**Figure 1:**
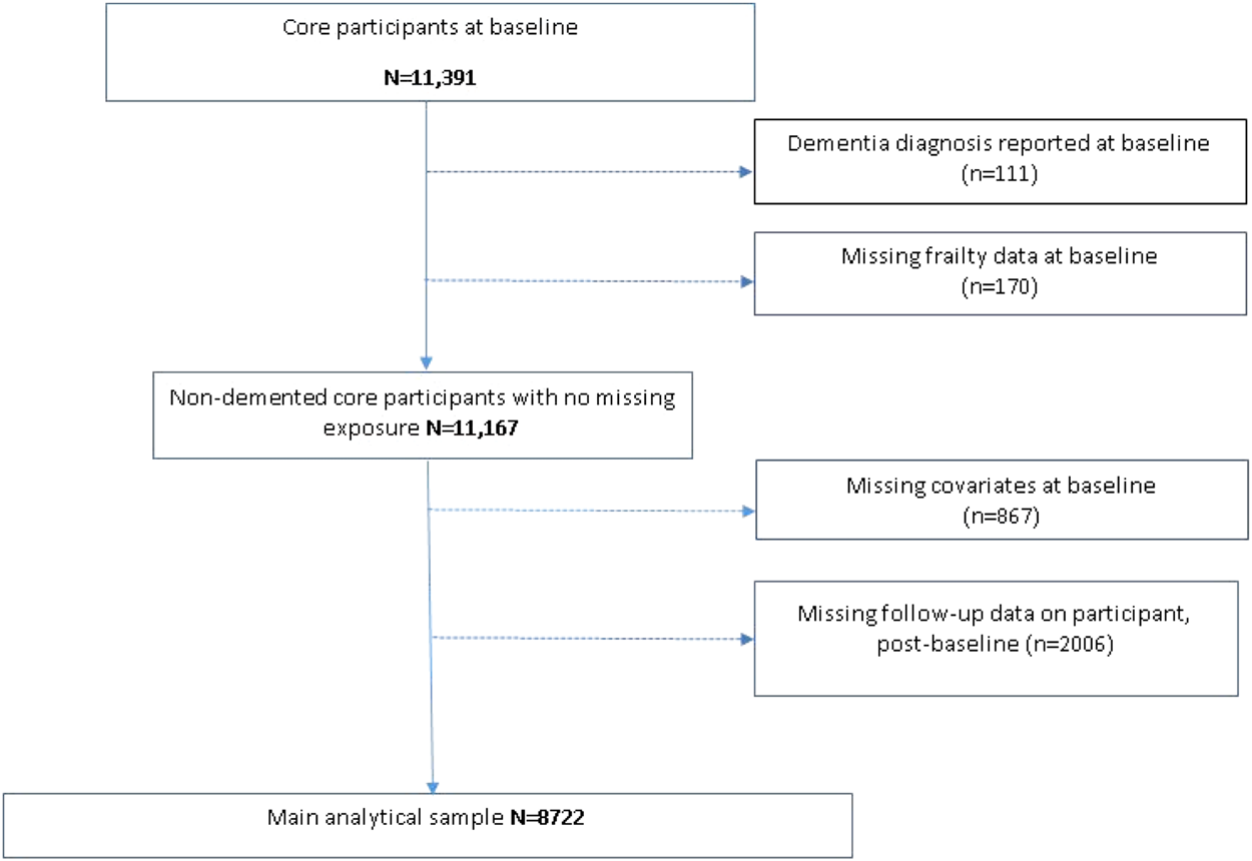
Flow diagram of study members into the analytical sample.

Out of the 8,722 eligible participants, 8,638 (99.0%) had non-missing data for all 47 variables, 60 (0.7%) had non-missing data for 46 variables, and the remaining 24 (0.3%) participants had non-missing data for between 39–45 variables). See Fig. 1 for a flow chart with participant sample selection.

### Outcome

#### Dementia incidence

The outcome was defined by incidence of self-report by the participant that a doctor had diagnosed them with dementia or Alzheimer’s disease. At each wave of ELSA, participants were asked the question “Has a doctor ever told you that you have (or have had) Alzheimer’s disease, dementia, organic brain syndrome, senility or any other serious memory impairment?”. For those who did not report an exact date for dementia diagnosis, we assumed the time for dementia onset as the mid-point between the two continuous waves (the one in which the participant reported no dementia and the successive wave where the participant reported a dementia diagnosis). Participants with a prevalent history of dementia at baseline were excluded from these analyses.

#### Mortality

Mortality data were used for those participants who had given written consent for linkage to official records from the National Health Service (NHS) central register. The month of death was recorded up until February 2013, at which point the study was right-censored.

#### Survival time

Participant age rather than study time was used as the time variable in the survival models. This was because the participants were all different ages at baseline and because there is a strong relationship between dementia and age^27^. Survival time was defined as age until the date of dementia diagnosis (the event of interest); death (competing-risk event), censoring (last date of contact) or the end of the study period, February 2013.

#### Covariates

To investigate any potential mechanisms that might explain the associations between frailty status and dementia, well-known factors associated with health outcomes were included as baseline covariates in all analyses. Covariates selected for inclusion in the analysis were age (years), sex (male or female), educational qualifications (no qualifications, O-levels or A-levels, degree/higher or equivalent), total non-pension wealth (quintiles), cohabitation (living with a partner/spouse or living alone), current smoking status (a smoker/non-smoker), alcohol consumption (drinking almost daily or not) and physical activity (mild, moderate or vigorous activity at least once a week or remaining sedentary)^25^.

#### Statistical analysis

Baseline differences between non-frail, pre-frail and frail adults were examined using means, standard deviations and t-tests for continuous variables and using means, percentages and Pearson’s chi-square tests for categorical variables. Competing risks regression models were used according to the methods of Fine and Gray^28^ and cumulative incidence function (CIF) models were used to predict the risk of participants developing dementia in relation to baseline frailty levels while taking into account death as a competing event^29^. Cox regression models were used alongside competing risks regression models to examine how taking account of competing risk of death influences the risk estimates associated with frailty and incidence of dementia. Cumulative incidence curves were produced to show the cumulative dementia risk over time. Cox proportional hazards regression models were used to test the relationship between frailty and mortality in the whole sample, whilst controlling for selected covariates. The Scheike and Zhang test was used to verify the assumption of proportional distribution hazards for each covariate^30^.

Separate analyses were run using global cognitive function scores (combined standardised scores on verbal fluency, immediate recall and delayed recall tests)^31^, which were dichotomised into two groups, lowest quartile of CF and upper three-quarters of global cognitive function. To explore if the cognitive function modifies the association between frailty and incident dementia, we derived an interaction term between frailty and cognitive function at baseline.

All analysis were performed using STATA 14 (StataCorp, LP, College Station, Texas).

## Results

The original sample at baseline comprised of 11,391 participants, 74 of whom reported a diagnosis of dementia and were excluded from the study. There were 10,522 participants who had non-missing data for baseline dementia status, all covariates and an adequate number of variables (30 or more out of a possible 47 variables) to generate a frailty index score. Of the 10,522 eligible participants, 8,722 (83%) had follow-up data post-wave 1, and these were included in the analytical sample (see Fig. 1). Of the 8,722 participants who were retained in the final analysis, 365 (4.2%) developed dementia, 1,455 (16.7%) died, and 6,902 (79.1%) were censored during the study period. The mean time to death or censoring was 9.4 (3.5) years in the total sample, 9.8 (SD: 3.4) years in the non-frail, 9.2 (SD: 3.5) years in the pre-frail and 8.3 (SD: 3.6) years in the frail.

Sample characteristics of the analytical sample are displayed in Table 1. The incidence of higher levels of frailty was greater in adults who were older, female, living alone, poorer and who had no educational qualifications.

**Table 1:** Demographic characteristics of the sample population, by frailty status (n = 8722).

	Full sample	Non-frail (n = 3679)	Pre-frail (n = 3572)	Frail (n = 1471)	Chi square test	P value
Frailty Index (FI) [mean (SD)]	0.14 (0.12)	0.04 (0.02)	0.15 (0.05)	0.38 (0.10)	—	—
Age [mean (SD)]	64.4 (9.8)	61.6 (8.5)	65.8 (9.8)	68.3 (10.7)	753.4	P < 0.0001
Female [n (%)]	4792 (54.9)	1739 (47.3)	2138 (59.9)	915 (62.2)	153.7	P < 0.0001
Wealth [n (%) in poorest quintile]	1521 (17.4)	327 (8.89)	647 (18.1)	547 (37.2)	882.2	P < 0.0001
No educational qualification [n (%)]	3477 (39.9)	1085 (29.5)	1516 (42.4)	876 (59.6)	470.0	P < 0.0001
Living alone [n (%)]	2670 (30.6)	811 (22.0)	1171 (32.8)	688 (46.8)	315.8	P < 0.0001
Cognitive function score [mean (SD)]	29.4 (8.4)	31.5 (8.03)	28.7 (8.16)	25.7 (8.12)	673	P < 0.0001
Consuming alcohol, daily basis [n (%)]	2502 (28.7)	1221 (33.19)	977 (27.4)	304 (20.7)	85.5	P < 0.0001
Current smoker [n (%)]	1,198 (13.7)	433 (11.7)	497 (13.9)	268 (18.2)	37.1	P < 0.0001
Sedentary [n (%)]	744 (8.5)	106 (2.88)	215 (6.02)	423 (28.8)	1900	P < 0.0001

Compared to adults who were non-frail, frail adults were approximately four times more likely to be in the poorest wealth quintile and nearly twice as likely to have no educational qualifications. Frail adults, compared with their less frail counterparts, were also more likely to smoke and to live a sedentary lifestyle, but they were less likely to drink alcohol on a daily basis. Frail adults were approximately ten times more likely to live a sedentary lifestyle than non-frail adults. The values of the FI closely fit a gamma distribution and had mean 0.14; median 0.01; and range from 0–0.78. The 99% limit of the FI was 0.56, which was the highest FI score that included 99% of observations.

Table 2 shows the hazard ratios (HRs) for incident dementia in each of the three frailty classes. Cox regression models were used in addition to competing risks regression models in order to explore how taking account of the competing threat of survival influences the risk estimates associated with frailty and incidence of dementia. The assumption of proportional distribution hazards for each covariate was found not to be violated (p-values ≥0.05).These results were generated in a stepwise multivariate model. After adjustment for sex and age (model 1), a status of pre-frail or frail compared to non-frail was associated with a significantly higher risk of developing dementia. Further adjustments for wealth, education and living alone (model 2) attenuated the associations between the degree of frailty and incident dementia although the association remained significant. Model 3 included all components of models 1 & 2, but additionally included alcohol intake, physical inactivity and smoking status. In model 3, the magnitude of the association between frailty status and developing dementia was further reduced, but remained significant in both adults who were pre-frail (competing risks regression HR: 1.51 [1.12–2.02]) and frail (competing risks regression HR: 1.73 [1.22–2.43]) when compared with adults who were non-frail. There was a positive association between the degree of frailty at baseline and incident dementia. Those who were frail at baseline consistently had a higher risk of developing dementia than those who were pre-frail. In the fully adjusted Cox regression model (equivalent to model 3), compared with a non-frail baseline status, there was a positive association between being pre-frail (HR: 1.60 [1.20–2.14]) or frail (HR: 1.93 [1.38–2.72]) and developing dementia. Compared with the Cox regression model, accounting for the competing risk of death in the fully adjusted models (Table 2; model 3) reduced the estimated association of incident dementia and being pre-frail and frail at baseline, by 6% and 10%, respectively.

**Table 2:** Frailty status* and the risk of incident dementia from competing risk models and Cox proportional hazards models (n = 8722).

	Competing risk model HR (95% CI)	Cox proportional hazard model HR (95% CI)
Model 1		
Non-frail	1.00 (reference)	1.00 (reference)
Pre-frail	1.60 (1.20–2.14)	1.74 (1.31–2.32)
Frail	2.10 (1.52–2.90)	2.53 (1.85–3.46)
Model 2		
Non-frail	1.00 (reference)	1.00 (reference)
Pre-frail	1.57 (1.17–2.09)	1.68 (1.26–2.24)
Frail	1.98 (1.42–2.76)	2.32 (1.69–3.19)
Model 3		
Non-frail	1.00 (reference)	1.00 (reference)
Pre-frail	1.51 (1.12–2.02)	1.60 (1.20–2.14)
Frail	1.73 (1.22–2.43)	1.93 (1.38–2.72)

*Frailty status: non-frail (n = 3679), pre-frail (n = 3572) and frail (n = 1471). Model 1: adjusted for sex and age. Model 2: adjusted for variables in model 1 plus wealth, educational qualifications and living alone. Model 3: adjusted for variables in model 2 plus alcohol intake, physical inactivity and smoking status.

### Survival

Cox proportional hazards regression models were used to compare the risk of mortality in individuals who were non-frail, pre-frail or frail. Compared with non-frail adults, those who were pre-frail (HR: 1.47 [1.28–1.68]) or frail (HR: 1.93 [1.64–2.27]) at baseline had a significantly reduced survival, after accounting for age, sex, wealth, educational qualifications, living alone, physical activity, alcohol intake and smoking. Compared with adults who were non-frail at baseline, similar HRs for dementia (Table 2) and death exist in adults who were pre-frail or frail at baseline.

**Figure 2:**
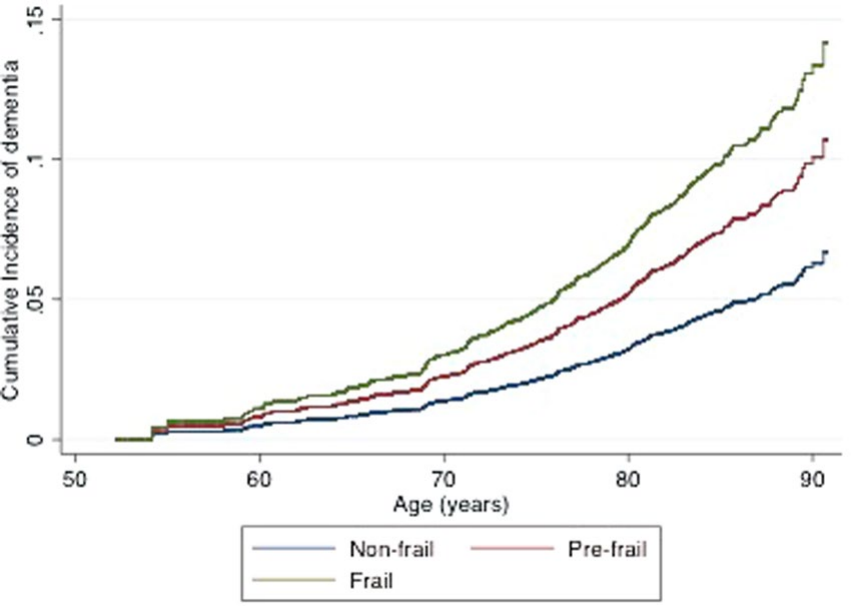
Modelled Cumulative incidence of dementia in baseline non-frail, pre-frail or frail adults, adjusted for all covariates and accounting for the competing risk of death.

Figure 2 shows the cumulative incidence of dementia (accounting for the competing risk of death and covariates) in adults who were non-frail, pre-frail and frail at baseline. The curves show that there are small but steadily increasing differences in the incidence of dementia between non-frail, pre-frail and frail adults, particularly between the ages of 50 and 70. From the age of approximately 70 and onwards, there is a much more rapid increase in the cumulative incidence of dementia in all three groups, but the steepest increase occurs in adults who were frail at baseline, followed by those who were pre-frail.

In the full sample, the relationship between frailty and incident dementia was found to be modified by cognitive function (P for interaction <0.0001). Hence, the relationship between frailty and dementia was further explored by taking account of baseline cognitive function. In participants with scores in the upper three quartiles of CF, compared to adults who were non-frail those who were pre-frail or frail had a significantly higher risk of dementia (Table 3). For adults in the lowest quartile of CF, when compared with the non-frail, being pre-frail or frail carried no increased risk of developing dementia.

**Table 3:** Frailty status (baseline) and risk of incident dementia, in adults in the lower quartile or upper three quartiles of cognitive function.

Lower quartile of CF (n = 2181)			Upper 3 quartiles of CF (n = 6541)		
	Competing risk model HR (95% CI)	Cox proportional hazard model HR (95% CI)		Competing risk model HR (95% CI)	Cox proportional hazard model HR (95% CI)
Non-frail (n = 595)	1.00 (reference)	1.00 (reference)	Non-frail (n = 3084)	1.00 (reference)	1.00 (reference)
Pre-frail (n = 973)	1.27 (0.89–1.83)	1.32 (0.92–1.88)	Pre-frail (n = 2599)	1.77 (1.09–2.89)	1.91 (1.16–3.14)
Frail (n = 613)	1.13 (0.74–1.71)	1.22 (0.81–1.84)	Frail (n = 858)	3.48 (1.98–6.12)	4.21 (2.35–7.55)

Risk of incident dementia is adjusted for sex, age, wealth, educational qualifications, living alone, alcohol intake, physical inactivity and smoking status.

## Discussion

In a population-representative sample of dementia-free older adults at baseline, we have shown that frailty, defined by an accumulation of health deficits, is associated with subsequent risk of dementia and death. This study revealed a dose-response relationship between the degree of baseline frailty severity and subsequent risk of dementia and death. To date, we are not aware of any other epidemiological studies that have taken into account the competing risk of death, when examining the relationship between frailty and incident dementia. In studies of older adults with long follow-up where mortality is high, a competing risk approach is recommended to reduce bias when calculating effect estimates^21^. We demonstrate in this study that the estimated risk of incident dementia in adults that are classified as pre-frail and frail can be made more accurate by taking account the competing risk of death.

Our results are also consistent with those of other studies, which report that frailty is an independent predictor of risk of dementia^5,9–12^. Song and colleagues derived a Frailty Index comprised of 19 health factors, which was found to independently predict dementia over a 5 and 10-year period even though none of the separate factors comprising the Frailty Index were individually associated with dementia^32^. The accumulation of health deficits appears to be characteristic of the ageing processes in humans with the increased acceleration of health deficits occurring in those with poor health^33^. Taking a life-course approach to improving the overall health of the population might, therefore, lessen the burden of dementia^34^. Focusing on a composite set of health risk factors in older adults (i.e. the Frailty Index) rather than single risk factor has been demonstrated to give a higher accuracy of predicting conditions of older age such as dementia^5^. Interestingly, dementia screening tools that include a multiple and diverse ranges of risk factors have been shown to be superior when compared to single factor models^35^. Similarly, the ability of the frailty index to predict dementia is improved by increasing the numbers of deficits considered as part of the frailty index^5^. Future studies are required to fully explore how thresholds of frailty severity affect the path of dementia progression.

It has been postulated that frailty and dementia might share a common aetiological pathway because both are associated with the rate of cognitive decline^36^. In this study, we show that for adults who were in the lowest quartile of CF at baseline, frailty severity was not associated with developing dementia. In contrast, adults in the upper three quartiles of CF at baseline were significantly more at risk of dementia if they were pre-frail or frail compared to non-frail. Our results resemble those of a previous study by Gray and colleagues^15^, who reported a link between frailty and incident dementia that was limited to those in the upper three quartiles of global cognitive functioning. Given that cognitive impairment is a strong predictor of dementia^37^ it is quite possible that adults in the lower quartile of global cognitive functioning were nearing dementia, leading to the obfuscation of any association between frailty and dementia.

The inclusion of a cognitive component to the frailty phenotype has been suggested by researchers because it has been shown to improve the predictive accuracy of frailty on clinical outcomes^38^. Consistent with other studies, our baseline data (Table 1) shows that there is a strong correlation between frailty and cognitive function^39^. Even in cases where cognitive impairment and frailty coexist, it is unclear whether their respective associations with dementia might involve the same mechanisms or whether they run along the same aetiological pathways. Whilst the mechanisms that link frailty to dementia are yet to be elucidated, evidence from clinical-pathologic studies revealed that the rate of progression of frailty was significantly associated with the accumulation of common brain pathologies including macro-infarcts, cerebrovascular disease, Alzheimer’s disease and Parkinson disease^40^. Risk factors that are common to both frailty and dementia include indicators of vascular dysregulation, hormonal systems, inflammation, insulin resistance, obesity and nutrition^41^. A deeper understanding of these systems is crucial to uncovering the mechanisms that might connect the two conditions. It has been suggested, for instance, that insulin resistance might increase the likelihood of becoming frail through acceleration of muscle protein degradation, which can ultimately lead to sarcopenia, a component of frailty^42^. There is also sound evidence that raised glucose levels are associated with future risk of dementia^43^. Individuals with metabolic syndrome and abdominal obesity, two markers associated with insulin resistance, are at increased risk of developing Alzheimer’s disease^44^. Work on rodents have shown direct interactions between insulin and amyloid-beta peptide signalling and crucially the ability of insulin to address the internalisation of amyloid-beta peptide, reducing its extracellular accumulation and potentially preventing amyloid-beta peptide toxicity from occurring^45^. The mechanisms and role of insulin resistance in dementia are complex and remain to be clarified in future studies. Like many chronic conditions of older age, dementia often progresses slowly via a preclinical stage, and it takes years before the clinical symptoms of dementia present themselves. This considerable ‘run-up’ period provides an opportunity to investigate factors that if altered before or during the preclinical period could be used as an intervention to prevent or delay the manifestation of dementia.

This study has several strengths including the use of a large representative study sample of older men and women and a prospective follow-up period of up to approximately ten years. Frailty was estimated using a frailty index that has been well validated in large epidemiological studies^18^. Importantly we were able to adjust our analysis for death as a competing event, enabling us to calculate unbiased and accurate cumulative dementia incidence. There are some limitations in this study including the lack of formal identification of dementia cases. Dementia cases in this study were identified through self-reported physician diagnosis. It was reported that in 2010/2011 just 42% of people living with dementia in England had received a formal diagnosis^4^ which may explain the lower number of dementia cases in this study compared with population prevalence estimates^46^ however,it might also suggest that the population sample is not entirely representative of the general population of England. Whilst we have taken into account the competing risk of death in the population sample we have not taken into consideration non-death attrition, which may result in an attenuation of the relationship between frailty and development of dementia.

## Conclusions

Increased levels of frailty are independently associated with an increased risk of dementia, although this was not observed in adults who have a low global cognitive function at baseline. These findings highlight that frailty should be considered alongside levels of cognitive performance when assessing the risk factors for dementia.

### Data availability

The datasets generated during the current study are available in the UK data service repository.

## Electronic supplementary material

Health deficits included in the frailty index and their values
