# Supplementary material for: Frailty is an independent predictor of incident dementia: Evidence from the English Longitudinal Study of Ageing
Source: Sci Rep. 2017 Nov 16;7:15746. doi: 10.1038/s41598-017-16104-y (PMC5691042; doi:10.1038/s41598-017-16104-y)
Supplement: Supplementary file 1 — Health deficits included in the frailty index and their values [file 41598_2017_16104_MOESM1_ESM.pdf]

Supplementary information

Frailty is an independent predictor of incident dementia: Evidence from the English Longitudinal Study of Ageing

Nina Rogers, Andrew Steptoe and Dorina Cadar

Domain	Frailty components						
Mobility Difficulties	Walking 100 yards	Yes =1	No =0				
	Sitting for about two hours	Yes =1	No =0				
	Getting up from a chair after sitting for long periods	Yes =1	No =0				
	Climbing several flights of stairs without resting	Yes =1	No =0				
	Climbing one flight of stairs without resting	Yes =1	No =0				
	Stooping, kneeling, or crouching	Yes =1	No =0				
	Reaching or extending arms above shoulder level	Yes =1	No =0				
	Pulling or pushing large objects like a living room chair	Yes =1	No =0				
	Lifting or carrying weights over 10 pounds, like a heavy bag	Yes =1	No =0				
	Picking up a 5p coin from a table	Yes =1	No =0				
Disability (ADL/iADL)	Dressing, including putting on shoes and socks	Yes =1	No =0				
	Walking across a room	Yes =1	No =0				
	Bathing or showering	Yes =1	No =0				
	Eating, such as cutting up your food	Yes =1	No =0				
	Getting in or out of bed	Yes =1	No =0				
	Using the toilet, including getting up or down	Yes =1	No =0				
	Using a map to figure out how to get around in a strange place	Yes =1	No =0				
	Preparing a hot meal	Yes =1	No =0				
	Shopping for groceries	Yes =1	No =0				
	Making telephone calls	Yes =1	No =0				
	Taking medication	Yes =1	No =0				
	Managing money, (e.g. paying bills and keeping track of expenses)	Yes =1	No =0				
	Doing work around the house or garden	Yes =1	No =0				
General health	Self-reported general health (fair/poor compared to excellent/very good/ good)	Excellent =0	V.good =0.25	Good = 0.5	Fair = 0.75	Poor = 1	
Depressive Symptoms	Respondent felt depressed much of the time during past week	Yes =1	No =0				

	Respondent felt that everything they did during the past week was an effort	Yes =1	No =0				
	Respondent felt that their sleep was restless during the past week	Yes =1	No =0				
	Respondent was not happy much of the time during the past week	Yes =1	No =0				
	Respondent felt lonely much of the time during the past week	Yes =1	No =0				
	Respondent did not enjoy life much of the time during the past week	Yes =1	No =0				
	Respondent felt sad much of the time during the past week	Yes =1	No =0				
	Respondent could not get going much of the time during the past week	Yes =1	No =0				
Self-reported conditions	High blood pressure or hypertension	Yes =1	No =0				
	Angina	Yes =1	No =0				
	Heart attack (including myocardial infarction or coronary thrombosis)	Yes =1	No =0				
	Congestive heart failure	Yes =1	No =0				
	An abnormal heart rhythm	Yes =1	No =0				
	Diabetes or high blood sugar	Yes =1	No =0				
	A stroke (cerebral vascular disease)	Yes =1	No =0				
	Chronic lung disease such as chronic bronchitis or emphysema	Yes =1	No =0				
	Asthma	Yes =1	No =0				
	Arthritis (including osteoarthritis, or rheumatism)	Yes =1	No =0				
	Osteoporosis, sometimes called thin or brittle bones	Yes =1	No =0				
	Cancer or a malignant tumour (excluding minor skin cancers)	Yes =1	No =0				
	Any emotional, nervous or psychiatric problems	Yes =1	No =0				
	Eyesight (while using lenses, if appropriate) poor compared to excellent	Excellent =0	V.good =0.2	Good = 0.4	Fair = 0.6	Poor = 0.8	Blind = 1
	Hearing (while using hearing aid, if appropriate) poor compared to excellent	Excellent =0	V.good =0.25	Good = 0.5	Fair = 0.75	Poor = 1	

Table S1: Health deficits included in the frailty index and their values
